# Evolutionary characteristics, biochemical structure, and function impact of *MSTN* gene

**DOI:** 10.1016/j.gendis.2025.101668

**Published:** 2025-05-02

**Authors:** Rui Zhang, Yunpeng Wu, Tianqi Sun, Zhengxing Lian, Jingqing Chen, Yefeng Qiu

**Affiliations:** a Academy of Military Medical Sciences, Beijing 100071, China; b Beijing Key Laboratory of Animal Genetic Improvement, Key Laboratory of Animal Genetics and Breeding of the Ministry of Agriculture, College of Animal Science and Technology, China Agricultural University, Beijing 100193, China

The myostatin (*MSTN*) gene, also known as growth and differentiation factor 8 (*GDF8*), plays a critical role in regulating muscle mass in animals by negatively controlling the number and size of skeletal myocytes. *MSTN* mutations have been demonstrated to cause the double-muscling (DBM) phenomenon in various species, including cattle, sheep, mice, pigs, dogs, rabbits, and even humans.^1^ In this study, we explored the evolutionary characteristics, biochemical structure and function impacts of the sheep *MSTN* gene (*oMSTN*) using phylogenetic analysis, mutation effect evaluation, residue conservation studies, structural modeling, and protein–protein docking. Our findings suggest that the evolutionary characteristics and biochemical structural features of *oMSTN* are closely tied to its functional and clinical roles in regulating skeletal muscle growth. We validated our hypothesis by creating *MSTN* gene-edited sheep using CRISPR/Cas9 technology. These results provide valuable insights for the preparation of animal models and the rapid and effective improvement of meat production. Furthermore, evaluating the effects of MSTN inhibition in animal models with diverse human diseases could support the development of MSTN inhibitors for future clinical applications.

Phylogenetic and evolutionary conservation analyses underscore the significant role of the “transforming growth factor beta (TGFβ)-like” domain in MSTN functionality. Phylogenetic studies have revealed that MSTN is closely related to GDF11 among the 11 GDFs and clustered into a single clade. As members of the activin/inhibin subclass, MSTN and GDF11 define a distinct subgroup within the larger TGFβ superfamily (Fig. 1A), consistent with previous phylogenetic analyses.^2^ The amino acid sequence of the mature MSTN protein, corresponding to the “TGFβ-like” domain, is conserved across species as divergent as humans and hooded crows (Fig. 1B). Residue conservation analysis using ConSurf, coupled with structural characterization, indicates that most amino acid positions in the TGFβ superfamily domain are highly conserved and evolve slowly (Fig. 1C). Functional predictions of MSTN mutations using PolyPhen-2, SIFT, and PROVEAN showed that mutations affecting MSTN functionality were concentrated in these highly conserved regions, likely disrupting polypeptide binding.

**Figure 1:**
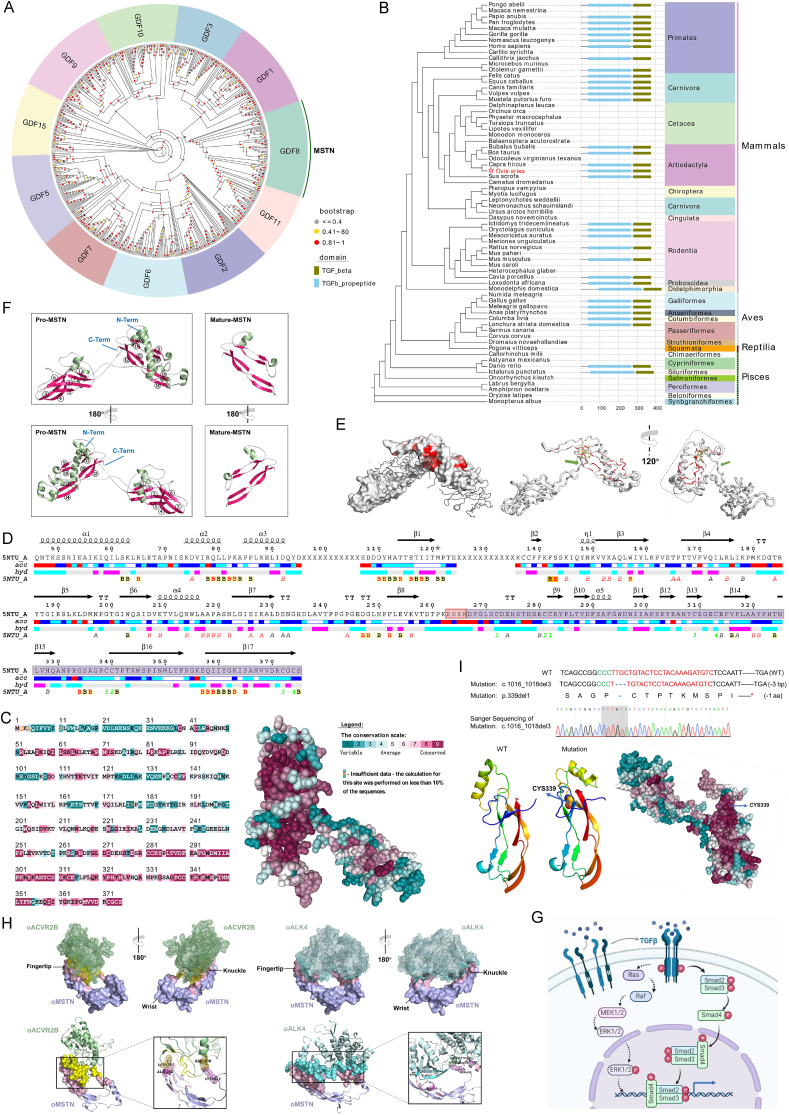
Evolutionary characteristics, biochemical structure features, and functions of oMSTN in regulating skeletal muscle growth. **(A)** Phylogenetic analysis of *MSTN* and other vertebrate *GDFs*. A maximum likelihood phylogenetic tree of all known vertebrate *GDFs* was constructed using their full-length sequences. Eleven *GDF* families are labeled in the tree, with each major family represented in a unique color. Some branches are collapsed for clarity. *MSTN* is phylogenetically related to *GDF11*, clustering into an individual clade. Together, MSTN and GDF11 form a distinct subgroup within the larger TGFβ superfamily. **(B)** Amplified *MSTN* clades of the phylogenetic tree. *oMSTN* is highly orthologous to goat *MSTN*. **(C)** Evolutionary conservation of *MSTN* amino acid positions. Conservation grades of amino acid positions in the o*MSTN* sequence and its 3D structure are displayed. Conservation levels range from the most variable positions (grade 1, turquoise) to the most conserved positions (grade 9, maroon). Positions with low-confidence conservation levels are marked in light yellow. The nine-color conservation scale is applied to the *oMSTN* sequence. **(D)** Predicted secondary structure of oMSTN. The secondary structure prediction of oMSTN, based on the pro-MSTN template (PDB ID: 5NTU), identifies 17 β-strands. The RSRR site and the C-terminal fragment are highlighted in light red and light purple, respectively. Structural features such as α-helices (medium squiggles), 3_10_ helices (small squiggles), π-helices (large squiggles), β-strands (arrows), strict α-turns (TTT), and β-turns (TT) are indicated in the secondary structure. **(E)** Sausage diagram of the latent oMSTN complex. Chain A and Chain B are visualized using a SOLV_SURFACE diagram and a gray Cα trace representation, respectively. Sequence conservation is displayed on a white-to-red color ramp. Disulfide bridges are shown as yellow sticks, the RSRR site is marked with a green arrow, and mature MSTN is enclosed in a dotted rectangle. **(F)** Ribbon diagram of oMSTN. The 3D structures of pro-MSTN and mature MSTN are displayed rotated 180° counterclockwise. α-helices are represented as green spirals, and β-strands are depicted as rose-red arrows. **(G)** MSTN signaling pathways. The mature MSTN dimer binds to ACVR2B, which recruits and activates ACVR1B/TβRI through transphosphorylation. Smad2 and Smad3 are subsequently activated, form a complex with Smad4, and translocate to the nucleus to regulate target gene expression. Aside from the canonical Smad-mediated pathway, mature MSTN can also activate Erk1/2 in the MAPK pathway via the Ras-MEK1 axis. **(H)** Prediction of oMSTN-receptor interactions. SOLV_SURFACE representations of oMSTN-oACVR2B and oMSTN-oALK4 heterodimeric interactions were generated through docking calculations. Both heterodimers are shown rotated 180° counterclockwise. Cartoon representations illustrate the heterodimerization interfaces, highlighting hydrogen bonds. oMSTN is shown in light blue, oACVR2B in pale green, and oALK4 in pale cyan. The heterodimerization interfaces of oMSTN-oACVR2B are shown in light pink and yellow, while those of oMSTN-oALK4 are shown in light pink and aquamarine. **(I)** Evolutionary changes and DBM phenotype in *MSTN* gene-edited sheep. Sanger sequencing detected modifications in *MSTN* loci of gene-edited sheep. PAM sequences and protospacer sequences are highlighted in green and red, respectively. Deletions (−) are marked in blue. A schematic diagram shows changes in the partial protein sequence of MSTN in knockout sheep, with deleted residues highlighted in blue. 3D models of wild-type and mutant MSTN proteins were constructed using the Phyre server, and the missing amino acid (CYS339) is highlighted in both 3D models with annotations from Phyre and ConSurf.

The function of oMSTN was further explored through secondary and tertiary structural predictions. The conserved nature of the “TGFβ-like” domain allowed us to model the secondary and tertiary structures of oMSTN using ENDscript (Fig. 1D, E) and the Phyre2 server (Fig. 1F), based on structural data from MSTN and other TGFβ family ligands. The oMSTN precursor protein consists of three distinct domains and undergoes two independent proteolytic processing events to become biologically active (Fig. S1). After proteolysis, the C-terminal dimer of MSTN remains within a latent complex with its propeptide and other proteins. Previous studies demonstrated that transgenic mice expressing the MSTN propeptide (amino acids 1–267) exhibited reduced MSTN activity, indicating that MSTN is negatively regulated by its propeptide. Additionally, other binding proteins, such as follistatin, can bind to the MSTN C-terminal dimer, inhibiting its receptor-binding ability.^2^ These findings suggest that overexpressing MSTN propeptide, in addition to MSTN knockouts, may serve as an alternative strategy for increasing muscle mass in mammals.

Protein–protein docking analyses were performed to investigate the interactions between oMSTN and its receptors. oMSTN likely signals via a mechanism analogous to TGFβ, involving the assembly of a hexameric signaling complex composed of the dimeric ligand, two type I receptors, and two type II receptors^3^ (Fig. 1G). Mature oMSTN interacts with cell surface receptors, including ALK4/ALK5 (type I receptors) and ACVR2A/ACVR2B (type II receptors) (Fig. 1H; Fig. S2), initiating specific intracellular signaling cascades via Smad2/3 proteins. Previous protein binding and crosslinking studies have shown that MSTN preferentially binds to ACVR2B. To explore this further, we predicted and analyzed oMSTN interactions with oACVR2B and oALK4, focusing on heterodimeric interfaces. Analysis with PDBePISA revealed that the binding interfaces were predominantly formed by residues in the highly conserved TGFβ superfamily region, highlighting its essential role in receptor binding.

The biochemical structures of the signaling domains in mature MSTN are critical to its function. As two highly similar members of the TGFβ family, earlier studies suggest that the ligands of mature MSTN and mature GDF11 are functionally indistinguishable, given their ability to bind to similar receptors and extracellular antagonists. However, MSTN has been recognized as a negative regulator of muscle growth and differentiation, whereas GDF11 is associated with beneficial effects on age-related dysfunction. Evidence supporting their functional divergence comes from genetic modifications in mice. Replacing the full coding region of MSTN with that of GDF11 produced mice lacking MSTN functionality, while substituting two specific amino acids in the fingertip region of GDF11 with corresponding MSTN residues yielded a phenotype consistent with GDF11-deficient mice.^4^ These findings demonstrate that structural and biochemical differences in the signaling domains of mature MSTN and GDF11 significantly contribute to their distinct roles in mammalian development and organ physiology.

As a critical gene underlying DBM, MSTN functionality has been strongly conserved across species. Increased muscling resulting from mutations in functional domains has been observed in cattle, sheep, and humans and in targeted mutations in mice, sheep, pigs, dogs, and rabbits. Mutations in MSTN's coding sequence alter its bioactivity, leading to DBM. For instance, the E291X mutation (a G-T transition) in Marchigiana cattle, the C313Y mutation (a G-A transition) in Piedmontese cattle, and an 11-nucleotide deletion at nucleotide 821 in Belgian Blue cattle all lead to DBM. In contrast, DBM in Texel sheep arises from a mutation in the 3′ untranslated region (G-A transition) of MSTN, which creates target sites for specific microRNAs. These microRNAs inhibit translation, reducing circulating MSTN levels. This observation highlights the need to incorporate non-coding region analysis into future MSTN research. Additionally, skeletal muscle hypertrophy in a 7-month-old boy was reported due to a splice-site mutation at the boundary of the first intron-exon of the human *MSTN* gene.^1^ This mutation disrupts MSTN transcription and causes premature termination, preventing translation of the bioactive domain.

To confirm our hypotheses, we generated *MSTN* gene-edited sheep with *FGF5* knockout using CRISPR/Cas9, creating *MSTN* double-allele knockout sheep. The resulting indels in *MSTN* led to the deletion of a highly conserved amino acid, significantly contributing to the DBM phenotype^5^ (Fig. 1I). *MSTN* promotes skeletal muscle myofiber hyperplasia through the MEK-ERK-FOSL1 axis.^5^

However, MSTN inhibition in primates, including humans, results in less pronounced muscle hypertrophy compared with mice. This disparity may stem from the non-uniqueness of MSTN in negatively regulating muscle mass through ACVR2B. Recent research identified activin A as a secondary molecule involved in the maintenance and growth of adult muscle mass. This discovery supports the combined use of MSTN and activin A antibody inhibitors to treat muscle atrophy and wasting disorders. These findings offer valuable insights and guide future directions for research and therapeutic strategies.

## CRediT authorship contribution statement

**Rui Zhang:** Conceptualization, Data curation, Formal analysis, Investigation, Methodology, Software, Visualization, Writing – original draft, Writing – review & editing. **Yunpeng Wu:** Methodology, Software, Visualization, Writing – review & editing. **Tianqi Sun:** Methodology, Software, Visualization. **Zhengxing Lian:** Conceptualization, Resources. **Jingqing Chen:** Conceptualization, Investigation, Validation, Writing – review & editing. **Yefeng Qiu:** Conceptualization, Funding acquisition, Project administration.

## Ethics declaration

All animal experimental protocols in this study were approved and supervised by the Animal Care and Use Committee at China Agricultural University. The study strictly adhered to the Guidelines for the Care and Use of Laboratory Animals issued by the National Institutes of Health.

## Conflict of interests

The authors declared no conflict of interests.

## Figures and Tables

**Fig.**
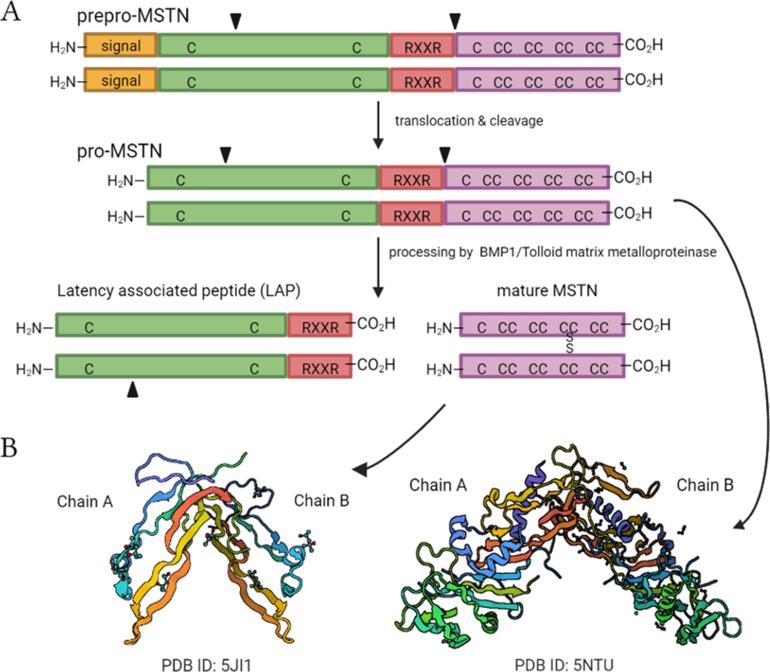
S1Proteolytic processing of MSTN protein. The first proteolytic processing removes the N-terminal (yellow) signal sequence, and the second generates the C-terminal fragment (light purple) by BMP-1/tolloid family of metalloproteinases. 3D structure of latent MSTN complex and mature-MSTN used the pro-MSTN (PDB ID: 5NTU) and the mature-MSTN (PDB ID: 5JI1) from human as the template, respectively.

**Fig. S2:**
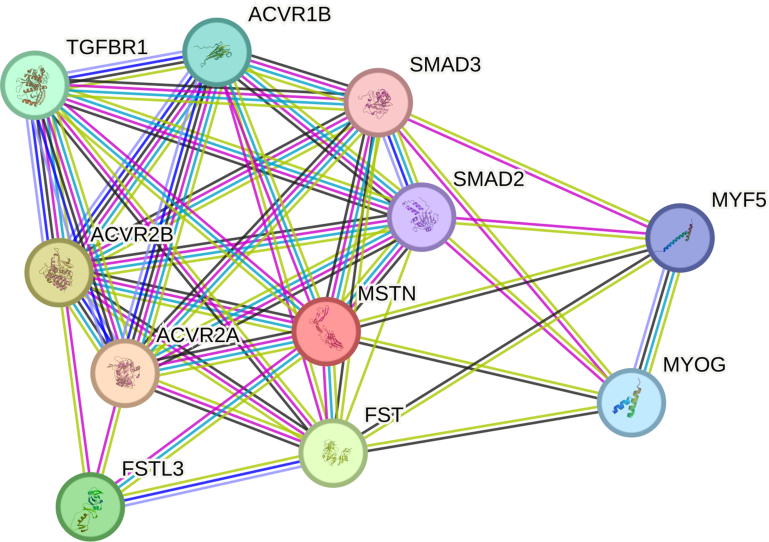
The MSTN signaling pathways. STRING database is used to search the interaction between MSTN and other proteins in sheep (*Ovis aries*). Interacting proteins were represented by nodes, and the interactions were represented by edges. The interactions between oMSTN and ALK4 (ACVR1B), ALK5 (TGFBR1), ACVR2A, or ACVR2B were all known interactions that were from both curated databases (showed by edges of baby blue) and experimentally determined (showed by edges of rose red).
