# Supplementary material for: Evolutionary characteristics, biochemical structure, and function impact of MSTN gene
Source: Genes Dis. 2025 May 2;12(6):101668. doi: 10.1016/j.gendis.2025.101668 (PMC12301908; doi:10.1016/j.gendis.2025.101668)
Supplement: Multimedia component 1 [file mmc1.docx]

Materials and Methods

Ethics Statement

All animal experimental protocols in this study were approved and supervised by the Animal Care and Use Committee at China Agricultural University. The study strictly adhered to the Guidelines for the Care and Use of Laboratory Animals issued by the National Institutes of Health.

Phylogenetic analysis of GDF genes

Protein sequences for vertebrate GDFs (GDF1 to GDF23) were retrieved from the NCBI database. Detailed species information and sequence identification numbers are provided in Supplementary Table 1. Pseudogene sequences were excluded. Multiple sequence alignment of vertebrate GDFs and MSTN full-length protein sequences was performed using MUSCLE in MEGA7. Gblocks was applied to refine the alignment by excluding poorly aligned or ambiguous columns, maximizing the alignment quality for analysis.

The maximum likelihood phylogenetic tree of GDFs was constructed using PhyML. The SMS server on the PhyML website was first used to identify the optimal substitution model (JTT+G+I+F with four substitution rate categories), which was then applied to tree construction. A separate maximum likelihood phylogenetic tree for MSTN was built using PhyML with the HIVb+G+I model and the same rate categories. Approximate likelihood ratio tests (aLRT SH-like) were performed to estimate branch support. Visualization and annotation of the GDF and MSTN phylogenetic trees were carried out using Evolview. Domain data for MSTN protein sequences were retrieved from PFAM.

Prediction of the functional eﬀects of MSTN mutations

The functional effects of MSTN mutations were assessed using PolyPhen-2 (<http://genetics.bwh.harvard.edu/pph2/>) and SIFT & PROVEAN v1.1.3 (http://provean.jcvi.org/), applying default cutoff values. PolyPhen-2 was used to predict the potential impact of amino acid substitutions on protein structure and function, while SIFT evaluated the effects of non-synonymous variants based on sequence homology. PROVEAN was employed to determine whether protein sequence variations altered functionality.

To identify function-altering mutations, multiple sequence alignments of MSTN proteins were analyzed across 68 species. Amino acid replacements occurring in the mature MSTN proteins were identified and evaluated using the web applications of PolyPhen-2, SIFT, and PROVEAN. The human MSTN protein sequence (NP_005250.1) was used as the query sequence. Mutations predicted as ‘possibly damaging’ by PolyPhen-2, ‘damaging’ by SIFT, or ‘deleterious’ by PROVEAN were considered to have functional significance.

Residue conservation analysis

The ConSurf algorithm was used to estimate the evolutionary conservation of amino acid residue positions within MSTN proteins. Multiple sequence alignments were generated using MAFFT, and the most suitable substitution matrix was selected. Conservation scores were computed under an empirical Bayesian paradigm to assign each residue a conservation grade.

A 3D model of the oMSTN protein was constructed using HHPred, which employs hidden Markov models to search for structural templates in the PDB. The MODELLER algorithm was subsequently applied to predict the 3D structure of the query sequence. Conservation scores ranged from grade 1 (highly variable, rapidly evolving residues) to grade 9 (highly conserved, slowly evolving residues). The conservation grades were visualized on the oMSTN sequence and its modeled 3D structure using a nine-color scale to highlight conservation levels.

Structural modeling

The secondary structure of MSTN was predicted using ESPript (https://espript.ibcp.fr) and ENDscript (<https://endscript.ibcp.fr>) from aligned sequences, and the results were showed using the oMSTN delimitations as the reference for other species.

For 3D structural modeling of oMSTN, I-TASSER , a hierarchical protein structure prediction method, and Phyre2, which uses a threading-based approach, were employed. These tools generated models based on templates derived from known structures in the Protein Data Bank (PDB). The resulting modeled structures were managed, visualized, and annotated using PyMol.

Protein–protein docking analysis

To investigate the interactions between oMSTN and its receptors, the structures of sheep activin A receptor type 2B (oACVR2B) and sheep ALK receptor tyrosine kinase (oALK4) were modeled using Phyre2. The modeled structures of oACVR2B and oALK4, along with oMSTN, were submitted to the ZDOCK server (https://zdock.umassmed.edu/), which evaluates all possible binding modes in translational and rotational space between two proteins. ZDOCK employs an energy-based scoring function to rank the predicted poses.

Further analysis of macromolecular interfaces for the oMSTN-oACVR2B and oMSTN-oALK4 complexes was performed using PDBePISA, an interactive tool for exploring protein-protein interaction surfaces.

Generation of MSTN knockout sheep via the CRISPR/Cas9 system

The CRISPR/Cas9 system was utilized to generate *MSTN* knockout sheep by directly injecting sgRNA and Cas9 mRNA into zygotes in a one-step procedure. The target site for *oMSTN* sgRNA was designed within the "TGF-beta-like" domain. Founders were identified through PCR amplification of the targeted *oMSTN* gene sequence, followed by sequencing to confirm the presence of induced mutations.

Structural models of oMSTN from both the founders and wild-type sheep were generated using Phyre2 to visualize and compare the structural changes resulting from the gene edits. The phenotypic effects of MSTN-null mutations, specifically the double-muscling (DBM) phenotype, were observed and documented in the knockout sheep.
