# Supplementary material for: Evolutionary characteristics, biochemical structure, and function impact of MSTN gene
Source: Genes Dis. 2025 May 2;12(6):101668. doi: 10.1016/j.gendis.2025.101668 (PMC12301908; doi:10.1016/j.gendis.2025.101668)
Supplement: Multimedia component 3 [file mmc3.docx]

Supplemental Table 2

| **Num** | [**oMSTN**](javascript:openWindow('pi_ipage_res2.html',400,250);) | [**HSDC**](javascript:openWindow('pi_ipage_hs.html',400,250);) | [**ASA**](javascript:openWindow('pi_ipage_asa.html',400,250);) | [**BSA**](javascript:openWindow('pi_ipage_bsa.html',400,250);) | [**Δ**^i^**G**](javascript:openWindow('pi_ipage_rdg.html',400,250);) |
| --- | --- | --- | --- | --- | --- |
| 16 | F:CYS 282 |  | 0.00 | 0.00 | 0.00 |
| 20 | F:LEU 286 |  | 85.37 | 4.85  \| | 0.08 |
| 22 | F:VAL 288 |  | 36.81 | 24.41  \|\|\|\|\|\|\| | 0.39 |
| 24 | F:PHE 290 |  | 18.17 | 2.66  \|\| | 0.04 |
| 25 | F:GLU 291 |  | 113.24 | 0.24  \| | -0.00 |
| 26 | F:ALA 292 | H | 84.61 | 32.60  \|\|\|\| | -0.26 |
| 27 | F:PHE 293 |  | 146.31 | 139.62  \|\|\|\|\|\|\|\|\|\| | 1.75 |
| 28 | F:GLY 294 |  | 55.82 | 27.61  \|\|\|\|\| | -0.05 |
| 29 | F:TRP 295 |  | 113.34 | 108.56  \|\|\|\|\|\|\|\|\|\| | 1.15 |
| 30 | F:ASP 296 |  | 116.89 | 31.55  \|\|\| | -0.36 |
| 31 | F:TRP 297 |  | 114.68 | 112.06  \|\|\|\|\|\|\|\|\|\| | 1.59 |
| 32 | F:ILE 298 |  | 9.08 | 3.02  \|\|\|\| | 0.05 |
| 38 | F:TYR 304 |  | 30.47 | 6.87  \|\|\| | -0.08 |
| 40 | F:ALA 306 |  | 8.99 | 3.35  \|\|\|\| | 0.05 |
| 65 | F:ASN 331 |  | 78.44 | 1.46  \| | -0.02 |
| 67 | F:LYS 333 | HS | 192.76 | 35.47  \|\| | -0.72 |
| 79 | F:MET 345 |  | 84.30 | 6.87  \| | 0.11 |
| 84 | F:MET 350 |  | 8.94 | 7.96  \|\|\|\|\|\|\|\|\| | 0.27 |
| 86 | F:TYR 352 |  | 36.70 | 18.95  \|\|\|\|\|\| | 0.26 |
| 87 | F:PHE 353 |  | 93.14 | 20.78  \|\|\| | 0.21 |
| 88 | F:ASN 354 |  | 48.02 | 24.62  \|\|\|\|\|\| | 0.08 |
| 89 | F:GLY 355 |  | 90.43 | 75.93  \|\|\|\|\|\|\|\|\| | 0.29 |
| 90 | F:LYS 356 |  | 131.69 | 16.06  \|\| | 0.26 |
| 91 | F:GLU 357 |  | 161.32 | 15.64  \| | 0.08 |
| 98 | F:ILE 364 |  | 55.01 | 37.51  \|\|\|\|\|\|\| | 0.60 |
| 99 | F:PRO 365 |  | 106.01 | 36.00  \|\|\|\| | 0.34 |
| 100 | F:GLY 366 |  | 37.59 | 14.54  \|\|\|\| | -0.17 |
| 101 | F:MET 367 |  | 44.63 | 37.30  \|\|\|\|\|\|\|\|\| | 1.09 |

**Interfacing residues of oMSTN in oMSTN-oACVR2B**

|  | Inaccessible residues |
| --- | --- |
|  | Solvent-accessible residues |
| HSDC | Residues making **H**ydrogen/**D**isulphide bond, **S**alt bridge or **C**ovalent link |
|  | Interfacing residues |
| **ASA** | Accessible Surface Area, Å² |
| **BSA** | Buried Surface Area, Å² |
| **Δ**^i^**G** | Solvation energy effect, kcal/mol |
| \|\|\|\| | Buried area percentage, one bar per 10% |
