# Supplementary material for: Evolutionary characteristics, biochemical structure, and function impact of MSTN gene
Source: Genes Dis. 2025 May 2;12(6):101668. doi: 10.1016/j.gendis.2025.101668 (PMC12301908; doi:10.1016/j.gendis.2025.101668)
Supplement: Multimedia component 4 [file mmc4.docx]

Supplemental Table 3

**Interfacing residues of oMSTN in oMSTN-oALK4**

| **Num** | **[oMSTN](javascript:openWindow('pi_ipage_res2.html',400,250);)** | [**HSDC**](javascript:openWindow('pi_ipage_hs.html',400,250);) | [**ASA**](javascript:openWindow('pi_ipage_asa.html',400,250);) | [**BSA**](javascript:openWindow('pi_ipage_bsa.html',400,250);) | [**Δ**^i^**G**](javascript:openWindow('pi_ipage_rdg.html',400,250);) |
| --- | --- | --- | --- | --- | --- |
| 16 | F:CYS 282 |  | 0.00 | 0.00 | 0.00 |
| 22 | F:VAL 288 |  | 38.67 | 2.68  \| | 0.04 |
| 24 | F:PHE 290 |  | 17.02 | 7.33  \|\|\|\|\| | 0.12 |
| 25 | F:GLU 291 |  | 113.69 | 13.21  \|\| | -0.14 |
| 26 | F:ALA 292 | H | 85.66 | 58.05  \|\|\|\|\|\|\| | 0.24 |
| 27 | F:PHE 293 |  | 146.59 | 81.85  \|\|\|\|\|\| | 0.86 |
| 28 | F:GLY 294 |  | 56.53 | 52.02  \|\|\|\|\|\|\|\|\|\| | 0.21 |
| 29 | F:TRP 295 |  | 114.10 | 112.10  \|\|\|\|\|\|\|\|\|\| | 1.20 |
| 30 | F:ASP 296 |  | 116.61 | 42.91  \|\|\|\| | -0.16 |
| 31 | F:TRP 297 |  | 115.11 | 106.45  \|\|\|\|\|\|\|\|\|\| | 1.48 |
| 32 | F:ILE 298 |  | 9.23 | 0.83  \| | 0.01 |
| 57 | F:HIS 323 |  | 89.85 | 21.17  \|\|\| | 0.22 |
| 59 | F:HIS 325 |  | 85.23 | 18.71  \|\|\| | -0.19 |
| 60 | F:LEU 326 |  | 118.47 | 57.73  \|\|\|\|\| | 0.84 |
| 61 | F:VAL 327 |  | 36.26 | 26.56  \|\|\|\|\|\|\|\| | 0.42 |
| 62 | F:HIS 328 |  | 103.71 | 29.92  \|\|\| | 0.35 |
| 63 | F:GLN 329 | H | 129.27 | 128.82  \|\|\|\|\|\|\|\|\|\| | -0.76 |
| 64 | F:ALA 330 |  | 88.90 | 87.06  \|\|\|\|\|\|\|\|\|\| | 0.62 |
| 65 | F:ASN 331 | H | 77.83 | 77.83  \|\|\|\|\|\|\|\|\|\|\| | -0.38 |
| 66 | F:PRO 332 |  | 99.01 | 43.49  \|\|\|\|\| | 0.70 |
| 67 | F:LYS 333 | HS | 193.24 | 70.24  \|\|\|\| | -0.73 |
| 68 | F:GLY 334 |  | 36.65 | 12.05  \|\|\|\| | 0.19 |
| 69 | F:SER 335 | H | 81.18 | 5.04  \| | -0.06 |
| 86 | F:TYR 352 |  | 38.39 | 14.30  \|\|\|\| | 0.23 |
| 87 | F:PHE 353 |  | 92.33 | 13.53  \|\| | 0.09 |
| 88 | F:ASN 354 |  | 48.55 | 28.58  \|\|\|\|\|\| | 0.11 |
| 89 | F:GLY 355 | H | 89.69 | 89.20  \|\|\|\|\|\|\|\|\|\| | 0.25 |
| 90 | F:LYS 356 |  | 131.83 | 28.94  \|\|\| | 0.46 |
| 91 | F:GLU 357 |  | 163.32 | 28.19  \|\| | 0.24 |
| 98 | F:ILE 364 |  | 54.55 | 19.57  \|\|\|\| | 0.31 |
| 101 | F:MET 367 |  | 43.78 | 13.43  \|\|\|\| | 0.49 |

|  | Inaccessible residues |
| --- | --- |
|  | Solvent-accessible residues |
| HSDC | Residues making **H**ydrogen/**D**isulphide bond, **S**alt bridge or **C**ovalent link |
|  | Interfacing residues |
| **ASA** | Accessible Surface Area, Å² |
| **BSA** | Buried Surface Area, Å² |
| **Δ**^i^**G** | Solvation energy effect, kcal/mol |
| \|\|\|\| | Buried area percentage, one bar per 10% |
